# The Maryland Dental Law Amendments

**Published:** 1886-04

**Authors:** 


### Editorial, Etc.

---

THE MARYLAND DENTAL LAW AMENDMENTS.—At the session of the State Legislature just closed, some important amendments were made to the dental law of this State, which renders it more liberal in its action, and more protective against the mountebanks and charlatans, who, for years past have infested the streets of our city on gilded chariots and with brass-band music.

The amendments relate to diplomas of reputable dental schools in other states as well as Maryland, being recognized, and the striking out of the illiteral provision of the old bill which restricted the practice of dentistry to graduates of the Maryland Dental Schools, or an examination by the Board of Dental Examiners; also the amendments prohibiting charlatans from going about the streets extracting teeth as an inducement for the purchase of quack medicines.

When the original bill containing the objectionable features recently removed from it was presented to the State Legislature for action, a much better bill had been suggested by the Maryland State Dental Association, against which a crusade was made, on account of its requiring an evidence of proficiency from all who had not been engaged in the practice of dentistry for ten years previously. The result of such an uncalled for opposition was the enacting of an illiteral bill, prepared in great part, we believe, by the judiciary committee of the House of Delegates, and to which the amendments have recently been made. A few more amendments, and this State law of Maryland will conform to the bill suggested by the State Society, an event to be desired, as then Maryland will have a proper dental law which will perfectly protect both the community and the dental profession. Had the bill as proposed by the State Society become the law, all the trouble attending the passage of the amendments would have been avoided. The instigators of the opposition are beginning to find out that it would have been better to have advocated, instead of opposed the State Association bill, and thus prevented the great blunder which required time and labor to correct.

